# Estimated Effectiveness of Nirsevimab Against Respiratory Syncytial Virus

**DOI:** 10.1001/jamanetworkopen.2025.0380

**Published:** 2025-03-10

**Authors:** Hanmeng Xu, Camila Aparicio, Aanchal Wats, Barbara L. Araujo, Virginia E. Pitzer, Joshua L. Warren, Eugene D. Shapiro, Linda M. Niccolai, Daniel M. Weinberger, Carlos R. Oliveira

**Affiliations:** 1 Department of Epidemiology of Microbial Diseases, Yale School of Public Health, New Haven, Connecticut; 2 Department of Pediatrics, Section of Infectious Diseases and Global Health, Yale School of Medicine, New Haven, Connecticut; 3 Department of Biostatistics, Yale School of Public Health, New Haven, Connecticut; 4 Department of Biostatistics, Section of Health Informatics, Yale School of Public Health, New Haven, Connecticut; 5 Department of Biomedical Informatics and Data Science, Yale School of Medicine, New Haven, Connecticut

## Abstract

**IMPORTANCE:**

Nirsevimab, a long-acting monoclonal antibody, demonstrated efficacy against respiratory syncytial virus (RSV)–associated lower respiratory tract infections (LRTI) in clinical trials. Postlicensure monitoring is essential to confirm these benefits in clinical settings.

**OBJECTIVE:**

To estimate the effectiveness of nirsevimab against medically attended RSV infections in infants and to assess how effectiveness varies by disease severity, dosage, and time since immunization.

**DESIGN, SETTING, AND PARTICIPANTS:**

This test-negative case-control study utilized inpatient, outpatient, and emergency department data from the Yale New Haven Health System. Nirsevimab-eligible infants who were tested for RSV using polymerase chain reaction between October 1, 2023, and May 9, 2024, were included. Infants with RSV-positive results were cases and infants with RSV-negative results were controls.

**EXPOSURE:**

Nirsevimab immunization, verified through state immunization registries.

**MAIN OUTCOMES AND MEASURES:**

Effectiveness was estimated using multivariable logistic regression, adjusting for age, calendar month, and potential confounders. Separate models examined estimated effectiveness by clinical setting, dosage, time since immunization, and severity (defined as needing high-flow oxygen or intensive care unit admission). Broader outcomes were also analyzed, including all-cause LRTI and all-cause LRTI-associated hospitalization.

**RESULTS:**

The analytic sample included 3090 infants (1722 male [57.3%]; median [IQR] age at testing, 6.7 [3.6-9.7] months), with 680 (22.0%) RSV-positive cases and 2410 (78.0%) RSV-negative controls. Nirsevimab uptake was 10.7% (330 patients), with 21 RSV-positive cases and 309 RSV-negative controls immunized. Adjusted effectiveness was 68.4% (95% CI, 50.3%-80.8%) against medically attended RSV infection, 61.6% (95% CI, 35.6%-78.6%) against outpatient visits, and 80.5% (95% CI, 52.0%-93.5%) against hospitalizations. The highest estimated effectiveness (84.6%; 95% CI, 58.7%-95.6%) was observed against severe RSV disease. Although estimated effectiveness against RSV infections declined from 79.3% (95% credible interval, 63.4%-90.6%) at 2 weeks postimmunization to 54.8% (95% credible interval, 16.3%-74.7%) at 14 weeks postimmunization, it remained significant. Estimated effectiveness did not vary substantially by dosage. During peak RSV season, nirsevimab appeared effective against all-cause LRTI (49.4%; 95% CI, 10.7%-72.9%) and all-cause LRTI-associated hospitalizations (79.1%; 95% CI, 27.6%-94.9%). From February to May 2024, when most LRTIs were caused by other viruses, its estimated effectiveness against these broader outcomes was negligible.

**CONCLUSIONS AND RELEVANCE:**

In this case-control study, nirsevimab provided substantial protection against RSV-associated outcomes. These findings support its continued use and provide evidence that may help boost public confidence in the immunization program.

## Key Points

**Question:**

What is the estimated effectiveness of nirsevimab against medically attended respiratory syncytial virus (RSV) infections in infants?

**Findings:**

In this test-negative case-control study with 680 RSV test-positive cases and 2410 RSV test-negative controls, nirsevimab’s estimated effectiveness was 68.4% against RSV infections, 80.5% against RSV-associated hospitalization, and 84.6% against severe RSV disease. Estimated effectiveness against RSV infection declined from 79.3% at 2 weeks postimmunization to 54.8% at 14 weeks postimmunization.

**Meaning:**

These findings suggest that nirsevimab provides protection against a wide range of RSV outcomes, but this diminishes over time, highlighting the need to optimize its implementation and sustain its uptake.

## Teaser

This case-control study estimates the effectiveness of nirsevimab against medically attended respiratory syncytial virus infections in infants by clinical setting, dosage, time since immunization, and severity.

## Introduction

Respiratory syncytial virus (RSV) is a major cause of acute lower respiratory tract infection (LRTI), particularly affecting newborns and infants. Globally, RSV is responsible for approximately 1.4 million hospitalizations and 13 300 in-hospital deaths annually among infants aged 0 to 6 months.^1^ The recent introduction of several prophylactic interventions provides a promising strategy to mitigate RSV’s impact on this vulnerable population.

Nirsevimab, a long-acting monoclonal antibody, was licensed by the US Food and Drug Administration in July 2023 after demonstrating safety and efficacy in prelicensure trials.^2^ These trials reported 79% efficacy against medically attended RSV, 81% efficacy against RSV requiring hospitalization, and 90% efficacy against severe RSV requiring intensive care unit (ICU) admission.^3^ Following its licensure, the Centers for Disease Control and Prevention Advisory Committee on Immunization Practices recommended nirsevimab for infants younger than 8 months entering their first RSV season and for high-risk infants and children aged 8 to 19 months.^4^

While prelicensure clinical trials demonstrated efficacy, it is essential to validate these findings through postlicensure studies that assess the effectiveness of nirsevimab in clinical settings. Such studies are needed to ensure that the protective effects of immunizations remain as they are being used in routine clinical practice, where factors like comorbidities, access to care, and clinician practices can influence outcomes. Early clinical data from the 2023 to 2024 RSV season in Europe and the US show effectiveness ranging from 70% to 90% against hospitalization for RSV-associated LRTI.^5,6,7,8,9^ However, several gaps in knowledge remain. Specifically, there are limited data on nirsevimab’s long-term effectiveness, its impact at different dosages, and its ability to prevent milder RSV cases. Furthermore, there is a need for further exploration of its effectiveness in diverse populations, particularly those with underlying health conditions. To address these gaps, this study aims to estimate the clinical effectiveness of nirsevimab in a diverse US patient population and examine how protection varies over time, by disease severity, and by dosage.

## Methods

### Study Design and Study Population

The effectiveness of nirsevimab against medically attended RSV infection was estimated using the test-negative case-control study design. The institutional review board at the Yale School of Medicine approved the study and waived the requirement for informed consent to due to its retrospective design and the minimal risk posed to participants. This study follows the Strengthening the Reporting of Observational Studies in Epidemiology (STROBE) reporting guidelines for observational studies.

The study population included all patients who were born after October 1, 2022, were tested for RSV due to a suspected acute respiratory infection, and received care in facilities affiliated with the Yale New Haven Health System (YNHHS) between October 1, 2023, and May 9, 2024. The YNHHS is the largest health system in Connecticut, and consists of 5 integrated hospital networks, 30 emergency or urgent care centers, and more than 130 outpatient clinics in Westchester County, New York; Rhode Island; and Connecticut, all integrated using a single electronic health record (EHR) system.

Patients were excluded if they were not age-eligible for nirsevimab when it became available on October 1, 2023,^10^ or if they resided outside Connecticut, New York, or Rhode Island. The geographic restriction was implemented to ensure that immunization records could be verified through state immunization registries, which are directly integrated with the YNHHS EHR. Infants were considered eligible for nirsevimab if they were born during the season (after October 1, 2023), if they were younger than 8 months and entering their first RSV season, or if they were between 8 and 12 months with at least 1 risk factor for severe RSV when entering the season. See eFigure 1 in Supplement 1 for the detailed inclusion process and eTable 1 in Supplement 1 for the definitions for risk factors.

### Data Sources and Study Definitions

For infants who met the eligibility criteria, reviews of medical records and state immunization registry searches were conducted to capture information on patient characteristics, immunization history, and potential confounders. Relevant clinical and laboratory data associated with each patient’s RSV test, such as chief complaints, problem lists, encounter diagnoses, and presence of any other acute or chronic diseases were abstracted by trained investigators from the EHR (see the eMethods in Supplement 1 for data abstraction details). The clinical outcomes following hospitalization were also recorded, such as hospital and ICU length of stay and maximum respiratory support needed during hospitalization. Patient characteristics including age, self-reported race and ethnicity, gestational age, birth weight, and type of insurance were also abstracted. Race and ethnicity categories included non-Hispanic Black, Hispanic, non-Hispanic White, and non-Hispanic other race (defined as American Indian or Native American, Asian, Middle Eastern or Northern African, and Pacific Islander); race and ethnicity were included given their known associations with immunization uptake and severe RSV outcomes. Individuals without documented evidence of a specific risk factor were assumed not to have it.

Cases were defined as infants with a medically attended RSV infection confirmed by nasopharyngeal polymerase chain reaction. Controls were infants with acute respiratory infection who tested negative for RSV. For a given patient, if there were multiple positive tests during the study period, only the first was included. If a patient had more than 1 negative test within 14 days, the first negative test was selected. If a patient had both positive and negative test results separated by 14 or more days, both records were retained because they represented distinct infection events. However, if the positive and negative results were within 14 days of each other, only the positive result was retained. The primary exposure of interest was the nirsevimab immunization status. Only documented immunization dates were included in the analysis. Infants were classified as immunized if they received a dose of nirsevimab prior to their RSV test.

### Statistical Analysis

#### Primary Analyses

The characteristics of the study population were summarized using frequency distributions and measures of central tendency. Missing data were either explicitly reported or included as a category within a relevant variable. Univariable analyses were performed to compare RSV-positive cases with negative controls, and unimmunized with immunized infants. Covariate balance between groups was assessed to detect potential confounders using standardized mean differences (SMD), with absolute SMDs of less than 0.20 indicating adequate balance.^11,12,13,14^

For our primary analysis, the effectiveness of nirsevimab against medically attended RSV infection was estimated using all eligible patients in our study population. Effectiveness was calculated as 1 minus the odds ratio of immunization with nirsevimab among cases and controls, using logistic regression. Noncollinear potential confounders were selected for the final adjusted models through backward selection based on the Akaike information criterion (eFigure 1 in Supplement 1). Missing data were addressed using listwise deletion. Due to collinearity between low birth weight and prematurity, as well as a high rate of missing data (approximately 25%), these variables were not used in the regression models. Instead, a composite variable indicating the presence of at least 1 risk factor for severe RSV disease was used (eTable 1 in Supplement 1). The adjusted effectiveness estimates presented are conditional estimates derived from multivariable models controlling for selected confounders. Model formulas and corresponding Akaike information criterion values are provided in eTable 2 in Supplement 1.

For our secondary aims, separate models were fitted to analyze the databased on clinical setting (inpatient vs outpatient), disease severity, nirsevimab dosage, and time from immunization. For the severity analysis, patients were considered to have severe disease if they were hospitalized within 14 days of RSV testing and required either transfer to a pediatric ICU or high levels of respiratory support during hospitalization, such as high-flow nasal cannula (≥2 L per minute), continuous or bilevel positive airway pressure, or invasive mechanical ventilation. The extent to which the estimated effectiveness of nirsevimab decreased over time was estimated using logistic regression within a Bayesian framework. The parameter representing the effectiveness of immunization was time-varying at biweekly intervals of time since immunization. These models used weakly informative prior distributions and imposed a monotonic structure on the regression coefficients that represent nirsevimab’s estimated effectiveness for increasing time since immunization. Posterior medians and 95% credible intervals were calculated from posterior samples, and convergence was evaluated using trace plots (eFigure 2 in Supplement 1). A comprehensive description of the Bayesian model is provided in the eAppendix in Supplement 1. The effectiveness of nirsevimab was also estimated using broader end points, including all-cause LRTI and all-cause LRTI hospitalization across the entire respiratory season (October 2023 to April 2024), with additional stratification by early (October 2023 to January 2024) and late (February to May 2024) periods.

#### Sensitivity Analyses

Several sensitivity analyses were conducted to assess the robustness of our findings. First, we assessed differences in estimated effectiveness when employing different exposure and outcome definitions. Specifically, we explored restricting our analysis to only medical visits where encounter diagnoses indicating LRTI were recorded as either a primary or secondary diagnosis (eTable 1 in Supplement 1). We also explored restricting controls to only those who tested positive for other respiratory viruses (ie, influenza, adenovirus, rhinovirus, and parainfluenza). In terms of exposure, we explored defining patients as immunized if they received nirsevimab 7 or more days prior to RSV testing, as was done in earlier reports to account for the mean RSV incubation period and the time required to reach peak antibody concentration.^9^ Second, we assessed whether excluding infants whose mothers received the maternal RSV vaccine or those who were born during the previous RSV season would significantly alter the results. Third, we repeated our analysis using the hepatitis B vaccine as a sham exposure, as previously described.^15,16^ Because the hepatitis B vaccine is recommended to be given to all newborns but does not affect the risk of RSV infection, we expect that in the absence of bias, the proportions of cases and of controls who were immunized with the hepatitis B vaccine will not be significantly different. Fourth, we applied an integrated nested laplace approximation model to examine temporal variation in estimated effectiveness. Fifth, we explored alternative modeling approaches, utilizing lasso regression and generalized estimating equation models, adjusting for multiple testing and prespecified covariates.

Further details on study definitions and statistical analysis are provided in eFigure 1, eTable 1, eTable 2, and eTable 3 in Supplement 1. All analyses were conducted in R, version 4.3.1.^17^ The threshold for statistical significance was a 2-sided *P* < .05.

## Results

### Study Population

**Figure 1.**
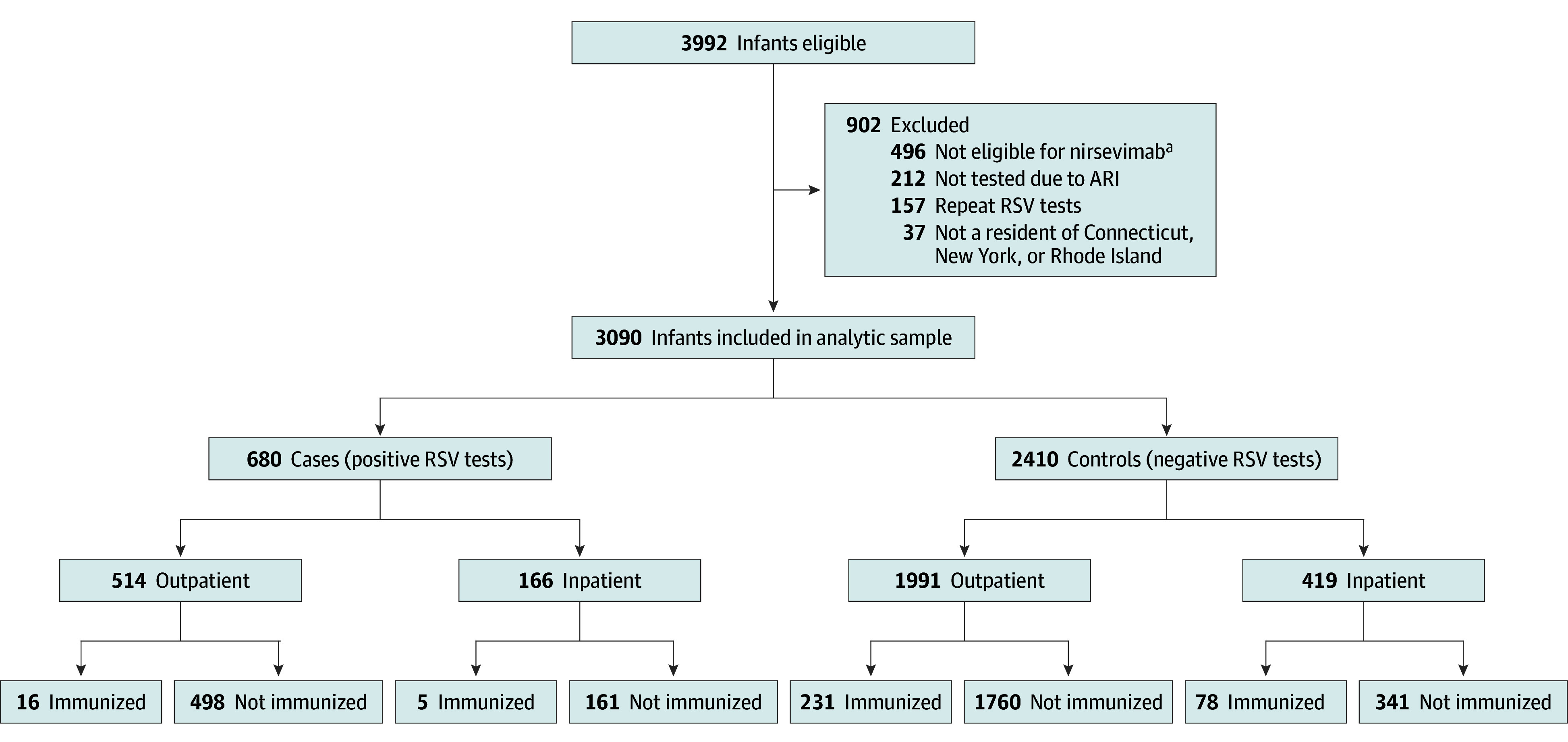
Selection Process of Respiratory Syncytial Virus (RSV) Test Records ARI indicates acute respiratory infection. ^a^Individuals aged older than 8 months on October 1, 2023, (when nirsevimab became available) but did not have risk factors for severe RSV disease.

Between October 1, 2023, and May 9, 2024, a total of 3090 RSV tests (1722 male [57.3%]; median [IQR] age at testing, 6.7 [3.6-9.7] months) were performed within the YNHHS that met our eligibility criteria and were included in the analysis (Figure 1). The analytic sample consisted of 680 patients (22.0%) with RSV-positive results and 2410 patients (78.0%) with RSV-negative results. Most RSV tests occurred during emergency department or urgent care clinic visits (2505 patients [81.1%]) between December 2023 and January 2024 (eFigure 3 in Supplement 1). RSV-positive cases were slightly younger than RSV-negative controls (median [IQR] age, 6.1 [3.4-9.2] months vs 6.9 [3.7-9.9] months; SMD = −0.14) and had a lower proportion of prematurity (76 cases [11.2%] vs 342 controls [14.2%]; SMD = 0.11). However, other demographic and clinical factors, including sex, race and ethnicity, insurance type, and prevalence of comorbidities, were comparable between the 2 groups (Table).

**Table.** Characteristics of Included Cases and Controls, October 1, 2023, to May 9, 2024

Characteristic	Participants, No. (%)			SMD^a^
	Overall (N = 3090)	Cases (n = 680)	Controls (n = 2410)	
Sex				
Female	1317 (42.6)	279 (41.0)	1038 (43.1)	0.05
Male	1772 (57.3)	401 (59.0)	1371 (56.9)	
Missing	1 (<0.1)	0	1 (<0.1)	
Age at testing, median (IQR), mo	6.7 (3.6-9.7)	6.1 (3.4-9.2)	6.9 (3.7-9.9)	−0.14
Race and ethnicity				
Hispanic	1328 (43.0)	280 (41.2)	1048 (43.5)	0.13
Black (non-Hispanic)	533 (17.2)	112 (16.5)	421 (17.5)	
White (non-Hispanic)	820 (26.)	201 (29.6)	619 (25.7)	
Other (non-Hispanic)^b^	161 (5.2)	26 (3.8)	135 (5.6)	
Unknown	248 (8.0)	61 (9.0)	187 (7.8)	
Birth weight				
Median (IQR), g	3214.3 (2824.9-3563.8)	3265.0 (2875.0-3576.2)	3194.5 (2805.1-3553.6)	0.10
Missing	783 (25.3)	184 (27.1)	599 (24.9)	
Gestational age, wk				
<37	418 (13.5)	76 (11.2)	342 (14.2)	0.11
≥37	1915 (62.0)	419 (61.6)	1496 (62.1)	
Missing	757 (24.5)	185 (27.2)	572 (23.7)	
Pulmonary diseases	156 (5.0)	26 (3.8)	130 (5.4)	0.07
Cardiac diseases	152 (4.9)	30 (4.4)	122 (5.1)	0.03
Anemia	94 (3.0)	15 (2.2)	79 (3.3)	0.07
Having at least 1 risk factor^c^	750 (24.3)	150 (22.1)	600 (24.9)	0.07
Insurance type				
Private	983 (31.8)	231 (34.0)	752 (31.2)	0.09
Public	2088 (67.6)	442 (65.0)	1646 (68.3)	
Uninsured	19 (0.6)	7 (1.0)	12 (0.5)	
Hospitalized				
Yes	585 (18.9)	166 (24.4)	419 (17.4)	0.17
No	2505 (81.1)	514 (75.6)	1991 (82.6)	
Nirsevimab status				
No	2760 (89.3)	659 (96.9)	2101 (87.2)	0.37
Yes, 100 mg dose	95 (3.1)	6 (0.9)	89 (3.7)	
Yes, 50 mg dose	235 (7.6)	15 (2.2)	220 (9.1)	

Abbreviation: SMD, standardized mean difference.

^a^ The difference in means between case and control participants in units of the pooled SD. Covariates with an absolute standardized mean difference greater than 0.2 were considered to have important imbalances.

^b^ Including American Indian or Native American, Asian, Middle Eastern or Northern African, and Pacific Islander by self-reporting.

^c^ Have at least 1 of the following conditions recorded in the infant’s medical history or diagnosis records: (1) anemia, (2) immunodeficiency (eg, transplantation history or leukemia), (3) cardiac diseases (including congenital heart diseases diagnosed at birth or any reporting of heart conditions), (4) pulmonary diseases, (5) Down syndrome, (6) small for gestational age (birth weight <2500 g), and (7) prematurity (gestational age <37 weeks).

The overall uptake of nirsevimab in the study sample was 10.7% (330 of 3090 patients), with 21 RSV-positive cases and 309 RSV-negative controls immunized. Uptake varied by hospital, ranging from 2.3% (9 of 399 patients) to 14.6% (200 of 1168 patients) (eTable 4 in Supplement 1). Among those who received nirsevimab before RSV testing, 235 (71.2%) received the 50 mg dose, while 95 (28.8%) received the 100 mg dose. Correlates of nirsevimab immunization are detailed in eTable 5 in Supplement 1. The uptake of the maternal RSV vaccine was 0.5% (14 of 3090 patients); among these infants, 6 also received nirsevimab. The median (range) interval between maternal vaccination and delivery was 16 (6-51) days.

Overall, 166 of 680 RSV-positive cases (24.4%) resulted in hospitalization. Among those hospitalized, 58.4% (97 of 166 patients) required more than 2 liters of respiratory support, and 13.8% (23 of 166 patients) required admission to the ICU (eTable 6 in Supplement 1).

### Estimated Effectiveness of Nirsevimab

**Figure 2.**
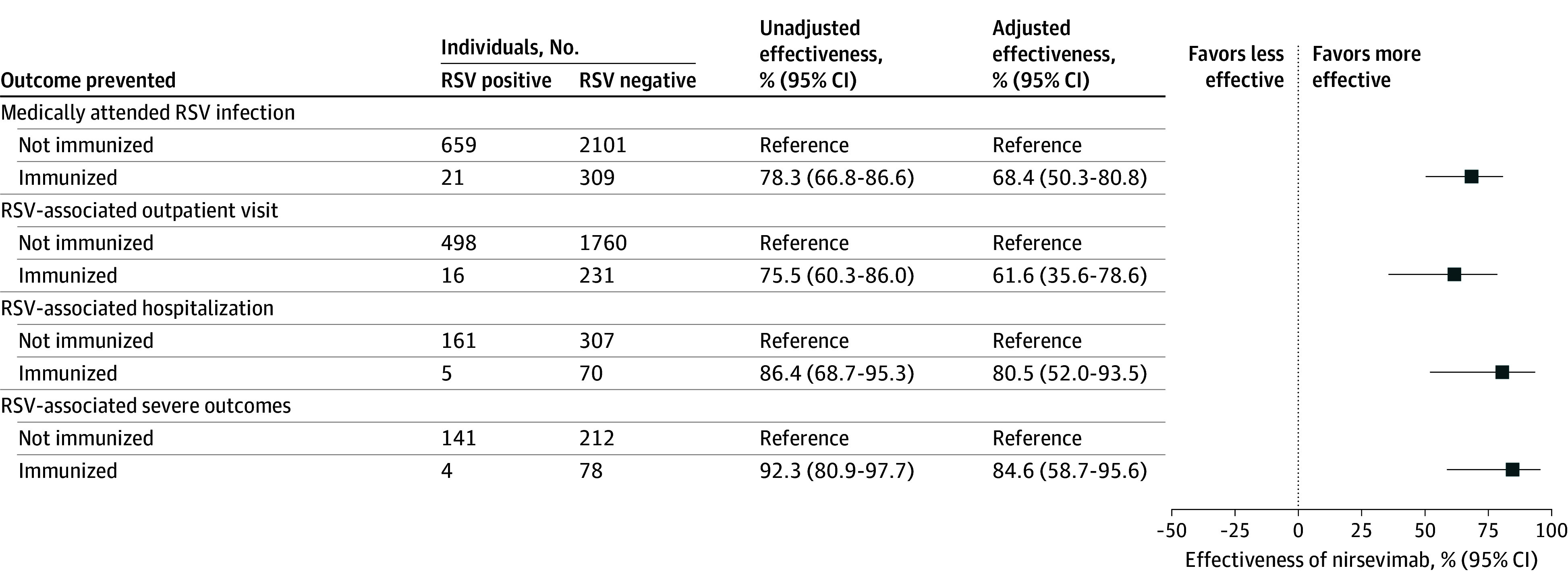
Estimated Effectiveness of Nirsevimab Against Medically Attended Respiratory Syncytial Virus (RSV) by Clinical Setting and Severity Adjusted models controlled for age, calendar month, and other potential confounders. Only hospitalizations and intensive care unit admissions with an admission date within 14 days of RSV testing were included in the analysis.

The adjusted effectiveness of nirsevimab against any medically attended RSV infection was 68.4% (95% CI, 50.3%-80.8%). Estimated effectiveness was 61.6% (95% CI, 35.6%-78.6%) for preventing RSV-associated outpatient visits, 80.5% (95% CI, 52.0%-93.5%) for preventing hospital admissions, and 84.6% (95% CI, 58.7%-95.6%) for preventing severe RSV (Figure 2). A comparison of these estimates with published clinical trials and observational studies is provided in eFigure 4 in Supplement 1. The dosage of nirsevimab did not significantly modify estimates of effectiveness (eFigure 5 in Supplement 1).

**Figure 3.**
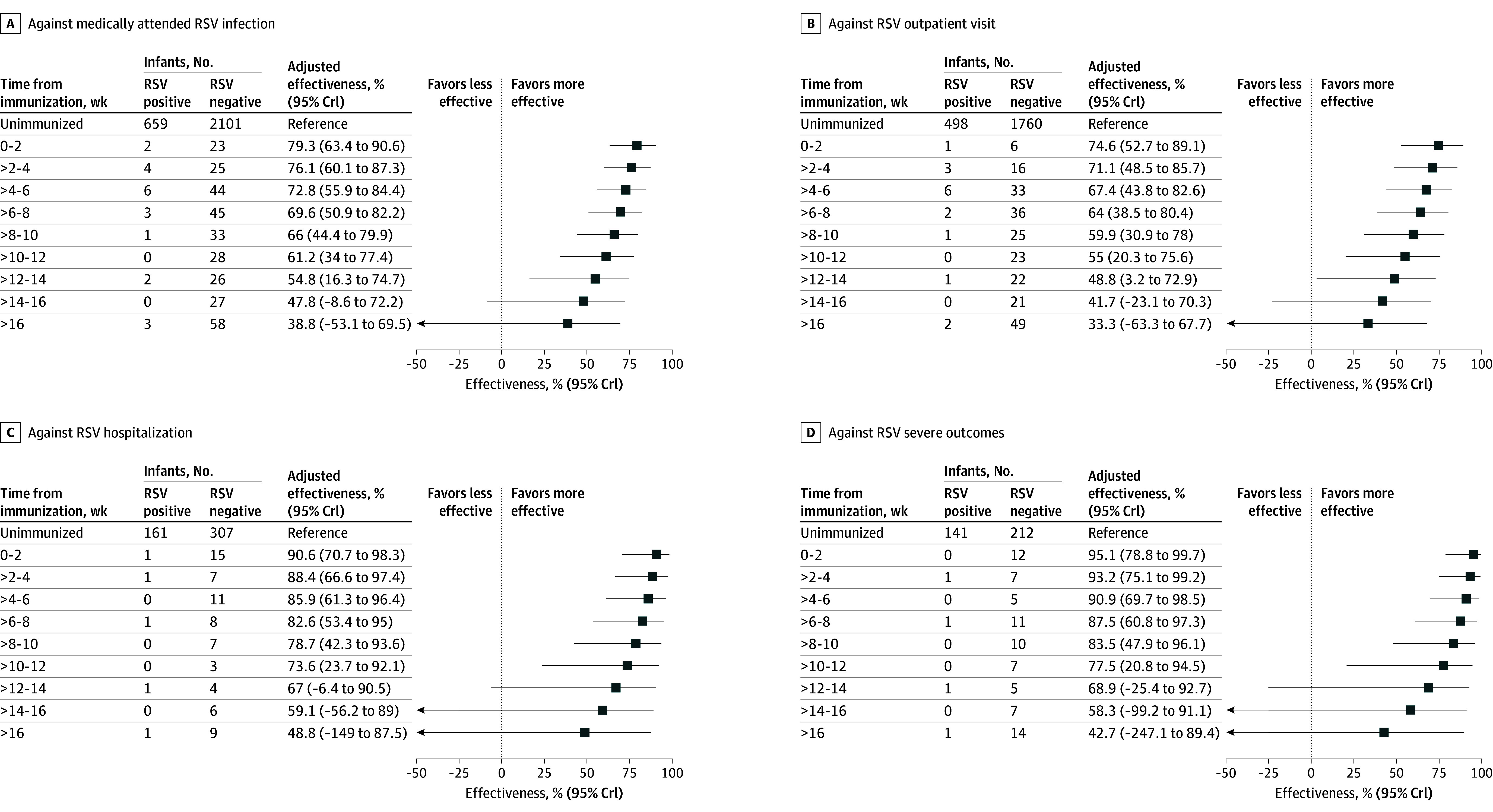
Estimated Effectiveness of Nirsevimab by Time Since Immunization, Estimated From a Bayesian Framework The boxes represent the median estimates of the effectiveness of nirsevimab in preventing various clinical outcomes (A-D), and the error bars indicate the 95% credible intervals (95% CrIs) of the estimates. The numbers in the second and third columns indicate the number of participants immunized a certain period of time before being tested for RSV.

The estimated effectiveness of nirsevimab waned over time, decreasing from 79.3% (95% credible interval, 63.4%-90.6%) at 2 weeks postimmunization to 54.8% (95% credible interval, 16.3%-74.7%) by 14 weeks post-immunization. This pattern of waning effectiveness was observed across all clinical outcomes (Figure 3) and was consistent with data from clinical trials (eFigure 6 in Supplement 1). Temporal effectiveness estimates relative to RSV activity in the community are shown in eFigure 7 in Supplement 1. Breakthrough RSV infections were most frequent between mid-November and December 2023, aligning with the peak RSV season. As RSV incidence declined later in the season, the number of breakthrough infections decreased, and estimated effectiveness appeared relatively higher.

**Figure 4.**
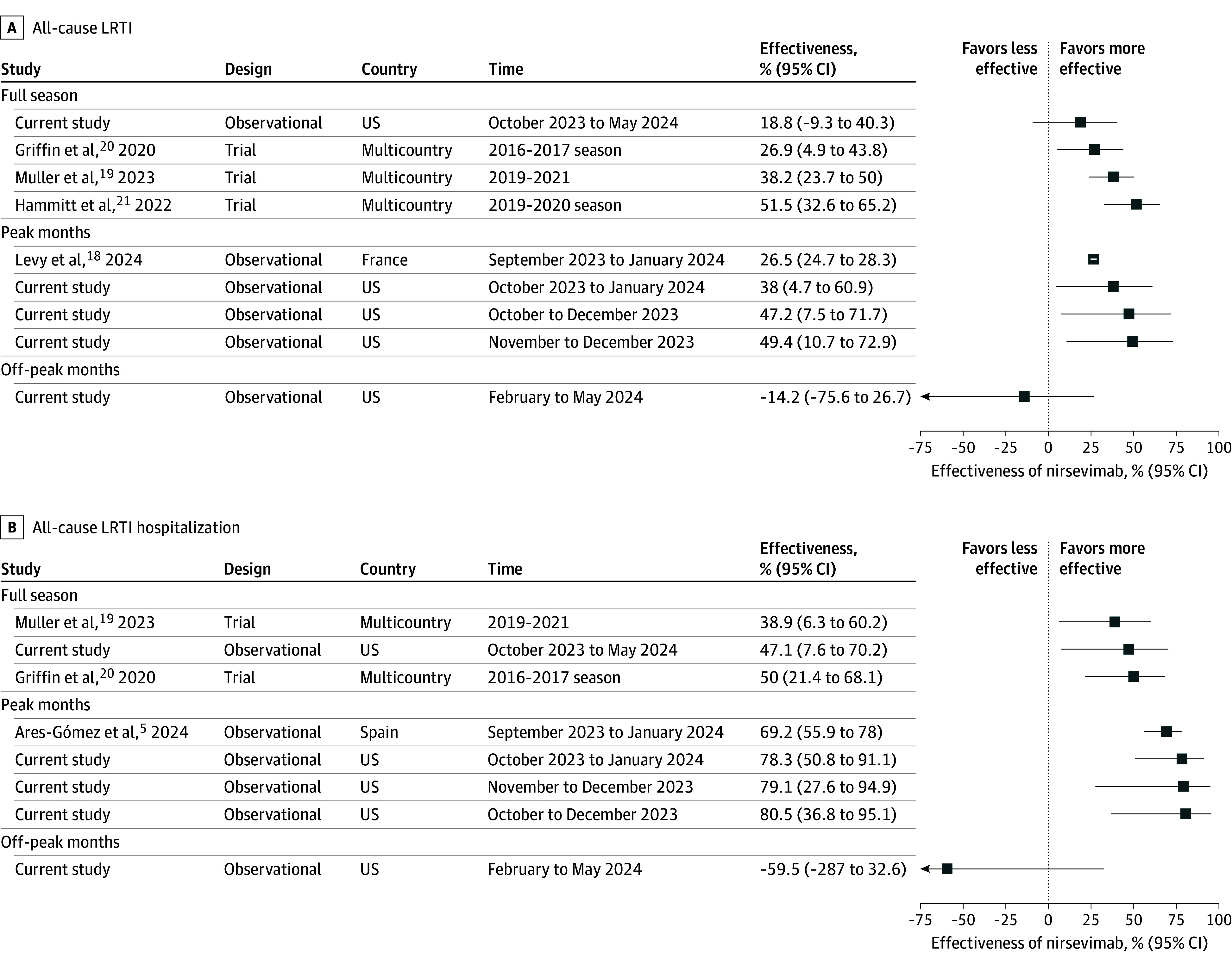
Nirsevimab Estimated Effectiveness Against All-Cause Lower Resipiratory Tract Infection (LRTI) and All-Cause LRTI Hospitalization, Stratified by Time For comparison with current study estimates, estimates from 5 previous studies^5,18,19,20,21^ are also included. Estimates were stratified by time (full season,^19,20,21^ peak months,^5,18^ and off-peak months). Only the estimate for the age group 3 to 12 months was shown for Levy et al.^18^ Estimates were adjusted for age, calendar time, presence of at least 1 risk factor for severe respiratory syncytial virus disease.

Protective effectiveness was observed against all-cause LRTI (49.4%; 95% CI, 10.7%-72.9%) and all-cause LRTI hospitalization (79.1%; 95% CI, 27.6%-94.9%) during the peak months of RSV season (November and December 2023) when compared with other studies^5,18,19,20,21^ (Figure 4). During these months, the RSV positivity rate was 39.3%. In contrast, between February and May 2024, when the RSV positivity dropped below 3.9% (eFigure 3 in Supplement 1), estimated effectiveness of nirsevimab against all-cause LRTI and all-cause LRTI hospitalizations was negligible (Figure 4). Results from additional post hoc explorative subgroup analyses are shown in eFigure 8 in Supplement 1.

All sensitivity analyses generated consistent results, with less than 10% differences in point estimates of effectiveness (eTable 6 in Supplement 1). As expected, the proportions of cases and controls that received the hepatitis B vaccine were nearly identical (365 of 680 cases [53.7%] and 1363 of 2410 controls [56.6%]; *P* = .18), and the corresponding estimated effectiveness of the hepatitis B vaccine against RSV was not statistically significant (eTable 3 in Supplement 1).

## Discussion

In this case-control study, we found robust evidence supporting the empirical benefits of nirsevimab, with an adjusted effectiveness of 68.4% against medically attended RSV infections. Our data indicate that estimated effectiveness was higher for RSV-associated hospitalizations (80.5%) and severe RSV disease (84.6%). These findings align with the prelicensure clinical trials, which reported 77% to 83% efficacy against RSV-associated hospitalizations.^22^ Emerging evidence from postlicensure studies, including a recent meta-analysis^23^ that estimated the effectiveness of 88.4% (95% CI, 84.7-91.2%) against RSV-associated hospitalizations, further supports the findings of this study.

Our study makes several important contributions to the existing literature. First, we measure the protective effect of nirsevimab in a diverse US patient population where historically minoritized racial and ethnic groups make up the majority (>50%) of the study sample; this is notable because most previous effectiveness estimates came from studies conducted primarily in European countries, which have distinct racial and ethnic compositions, different socioeconomic contexts, and considerably higher nirsevimab coverage. For instance, 15 of 16 postlicensure effectiveness studies to date have been conducted in Western Europe,^5,6,7,8,18,24,25,26,27,28,29,30,31,32,33^ where nirsevimab coverage in the target population often exceeded 70%.

Second, previous analyses, including the only US-based report,^9^ have primarily focused on RSV-associated hospitalizations, with limited evaluation of effectiveness against medically attended outpatient visits, which represent a substantial portion of the RSV burden.^34,35^ Our study addresses this gap and also extends the evaluation of nirsevimab to its impact on broader outcomes, such as all-cause LRTI. Our effectiveness estimates against all-cause LRTI and all-cause LRTI hospitalization during the peak months of the RSV season were comparable with estimates from other studies (Figure 4). Given that RSV was the predominant virus during these months (RSV positivity rate, 39.3% in our sample), these estimates largely reflect the effectiveness of nirsevimab against RSV. Prior postlicensure studies reported effectiveness of 69.2% (95% CI, 55.9-78.0%) against all-cause LRTI hospitalizations,^5^ nearly double the 39% efficacy observed in phase III trials against these nonspecific outcomes.^19^ Our findings align more closely with clinical trial data, and, notably, we found no significant protective effect against all-cause LRTI outside the peak RSV season when a negligible proportion of LRTIs were due to RSV.

Third, our study provides valuable insights into the temporal dynamics of nirsevimab’s estimated effectiveness. Given that our study spanned the entire RSV season (October 2023 to April 2024), we were able to assess how nirsevimab’s estimated effectiveness wanes over time—a factor less emphasized in earlier studies with shorter observation periods. While effectiveness appeared to decline over time, the lower rates of illness and hospitalization remained statistically significant for at least 14 weeks postimmunization. This waning pattern is consistent with what is known about the pharmacokinetics of monoclonal antibodies and the natural decay of passive immunity.^36^ The observed decline in effectiveness beyond 14 weeks, although preliminary and based on estimates with wide uncertainty intervals, underscores the importance of timing in administering nirsevimab, especially in regions with prolonged RSV seasons.

### Limitations

Our study has several limitations. First, the prioritization of high-risk infants for immunization during the early roll-out phase may have introduced confounding by indication.^37^ However, we conducted several sensitivity analyses that suggest residual confounding by unmeasured factors is less likely. Second, although our study benefited from a large sample size, the low uptake of nirsevimab resulted in limited statistical power and wide confidence intervals for certain comparisons, such as effectiveness by dosage. Third, because few cases were immunized more than 14 weeks before RSV testing, the effectiveness estimates beyond this period have wide credible intervals and should be interpreted with caution. Additionally, our secondary analyses were exploratory and not adjusted for multiple comparisons; future studies with prespecified hypotheses are needed to confirm these findings. Fourth, incomplete documentation within the EHR may have led to underestimation of certain risk factors. Fifth, underascertainment of immunization or prior infections may have biased our results toward the null. However, the uptake of nirsevimab in our study is very similar to that reported by other US-based studies and Centers for Disease Control and Prevention coverage estimates for Connecticut (7.7% uptake).^9,38^ Sixth, while all infants hospitalized with acute respiratory infection were tested for RSV as part of the YNHHS infection prevention protocols, outpatient RSV testing criteria were not as standardized. Consequently, testing decisions may have been influenced by the patients’ prior immunization status. To evaluate any potential residual confounding due to differences in testing practices, we employed a negative control exposure approach using a sham vaccine, as previously described.^39,40^ The absence of an association between the sham vaccine and RSV infection supports the validity of our findings and suggests that residual confounding likely had minimal impact on our estimates of effectiveness.

## Conclusions

In this test-negative case-control study, nirsevimab was highly effective at preventing RSV-associated outpatient visits, hospitalizations, and severe diseases requiring ICU admission or high-flow oxygen. Its estimated effectiveness persisted for at least 3 months postvaccination, consistent with results from randomized trials. These findings reinforce the benefits of RSV immunoprophylaxis and support US guidelines recommending nirsevimab for all infants entering their first RSV season.

## Notes

Supplement 1. **eFigure 1.** Correlation Between Potential Confounders in the Test-Negative Case-Control Analysis **eTable 1.** Definition of Key Clinical Outcomes and Risk Factors **eMethods.** Data Abstraction From Electronic Medical Records **eTable 2.** Variable Selection for the Multivariable Logistic Regression Models **eFigure 2.** Trace Plots for the Coefficients of Waning Effectiveness **eAppendix.** Estimating the Effectiveness of Nirsevimab by Time Since Immunization **eTable 3.** Sensitivity Analysis **eFigure 3.** RSV Tests and Nirsevimab Doses During the Study Period **eTable 4.** Comparison of Included Records by Hospital Visited **eTable 5.** Comparison of Immunized and Unimmunized Patients **eTable 6.** Clinical Characteristics of RSV-Positive Cases **eFigure 4.** Overview of Nirsevimab Effectiveness: Current Study Estimates in Context With Prior Research **eFigure 5.** Effectiveness of Nirsevimab Against RSV Infections by Dose, Clinical Setting, and Disease Severity **eFigure 6.** Effectiveness of Nirsevimab Against RSV-Associated LRTI by Time Since Immunization **eFigure 7.** Effectiveness of Nirsevimab Against Medically Attended RSV Over Calendar Time **eFigure 8.** Subgroup Analysis of Nirsevimab Effectiveness Against Medically Attended RSV Infection
eReferences.

Supplement 2.
Data Sharing Statement
