# Errors in Results, Figures, and Supplement 1

**DOI:** 10.1001/jamanetworkopen.2025.12578

**Published:** 2025-04-23

In the Original Investigation titled “Estimated Effectiveness of Nirsevimab Against Respiratory Syncytial Virus,”^1^ published March 10, 2025, there were errors in the results. First, in the Results section of the Abstract and second section of the Results, the 2 point estimates referring to the waning of nirsevimab’s estimated effectiveness over 2 weeks should have included 95% credible intervals (CrIs) rather than 95% CIs. Second, in Figure 1, the number of outpatient controls who were not immunized was reported as 1769 instead of 1760. Third, in Figure 2, a clarifying statement was added to the caption stating that only hospitalizations and intensive care unit admissions with an admission date within 14 days of respiratory syncytial virus testing were included in the analysis. Fourth, in Figure 3, the measure of variance was reported as a 95% CI instead of a 95% CrI. Finally, the same clarifying statement as Figure 2 was added to eTable 4 and eFigure 5 in Supplement 1. This article has been corrected.^1^
