# Supplementary material for: Estimated Effectiveness of Nirsevimab Against Respiratory Syncytial Virus
Source: JAMA Netw Open. 2025 Mar 10;8(3):e250380. doi: 10.1001/jamanetworkopen.2025.0380 (PMC11894488; doi:10.1001/jamanetworkopen.2025.0380)
Supplement: Supplement 1. — eFigure 1. Correlation Between Potential Confounders in the Test-Negative Case-Control Analysis eTable 1. Definition of Key Clinical Outcomes and Risk Factors eMethods. Data Abstraction From Electronic Medical Records eTable 2. Variable Selection for the Multivariable Logistic Regression Models eFigure 2. Trace Plots for the Coefficients of Waning Effectiveness eAppendix. Estimating the Effectiveness of Nirsevimab by Time Since Immunization eTable 3. Sensitivity Analysis eFigure 3. RSV Tests and Nirsevimab Doses During the Study Period eTable 4. Comparison of Included Records by Hospital Visited eTable 5. Comparison of Immunized and Unimmunized Patients eTable 6. Clinical Characteristics of RSV-Positive Cases eFigure 4. Overview of Nirsevimab Effectiveness: Current Study Estimates in Context With Prior Research eFigure 5. Effectiveness of Nirsevimab Against RSV Infections by Dose, Clinical Setting, and Disease Severity eFigure 6. Effectiveness of Nirsevimab Against RSV-Associated LRTI by Time Since Immunization eFigure 7. Effectiveness of Nirsevimab Against Medically Attended RSV Over Calendar Time eFigure 8. Subgroup Analysis of Nirsevimab Effectiveness Against Medically Attended RSV Infection eReferences. [file jamanetwopen-e250380-s001.pdf]

Supplemental Online Content

Xu H, Aparicio C, Wats A, et al. Estimated effectiveness of nirsevimab against respiratory syncytial virus. *JAMA Netw. Open*. 2025;8(3):e250380. doi:10.1001/jamanetworkopen.2025.0380

eFigure 1. Correlation Between Potential Confounders in the Test-Negative Case-Control Analysis
eTable 1. Definition of Key Clinical Outcomes and Risk Factors
eMethods. Data Abstraction From Electronic Medical Records
eTable 2. Variable Selection for the Multivariate Logistic Regression Models
eFigure 2. Trace Plots for the Coefficients of Waning Effectiveness
eAppendix. Estimating the Effectiveness of Nirsevimab by Time Since Immunization
eTable 3. Sensitivity Analysis
eFigure 3. RSV Tests and Nirsevimab Doses During the Study Period
eTable 4. Comparison of Included Records by Hospital Visited
eTable 5. Comparison of Immunized and Unimmunized Patients
eTable 6. Clinical Characteristics of RSV-Positive Cases
eFigure 4. Overview of Nirsevimab Effectiveness: Current Study Estimates in Context With Prior Research
eFigure 5. Effectiveness of Nirsevimab Against RSV Infections by Dose, Clinical Setting, and Disease Severity
eFigure 6. Effectiveness of Nirsevimab Against RSV-Associated LRTI by Time Since Immunization
eFigure 7. Effectiveness of Nirsevimab Against Medically Attended RSV Over Calendar Time
eFigure 8. Subgroup Analysis of Nirsevimab Effectiveness Against Medically Attended RSV Infection
eReferences

This supplemental material has been provided by the authors to give readers additional information about their work.

© 2025 Xu H et al. *JAMA Network Open*

eFigure 1. Correlation Between Potential Confounders in the Test-Negative Case-Control Analysis

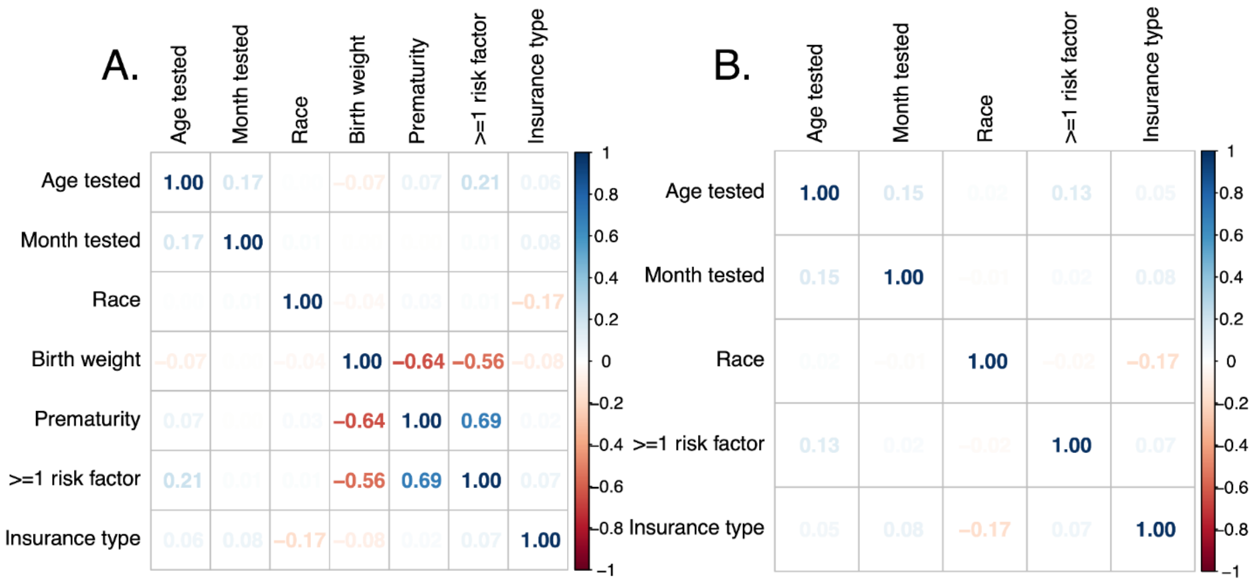

Multivariate logistic regression was used to estimate nirsevimab effectiveness against various clinical outcomes. Potential confounders were selected using backward selection from variables in the initial model (panel A), including age at testing (<3, 3-5, 6-8, 9-11, ≥12 months), calendar month of testing, race/ethnicity, birth weight, prematurity (gestational age <37 weeks), presence of at least one risk factor (see eTable 1), and insurance type (private, public, uninsured). The numbers show the correlation coefficients between any of the two variables, and a value larger than 0.5 was defined as a moderate or strong correlation. Due to collinearity between low birth weight and prematurity, and high rates of missing data (~25%), only the "at least one risk factor" variable was retained (panel B).

© 2025 Xu H et al. *JAMA Network Open*

eTable 1. Definition of Key Clinical Outcomes and Risk Factors

Acute respiratory illness (ARI)	Risk factors for severe RSV diseases	Medically attended upper respiratory infections (URTI)	Medically attended lower respiratory infections (LRTI)
Acute onset (<10 days) illness that presents with at least two of the following: fever (measured or subjective), chills, rigors, myalgia, headache, sore throat, nausea or vomiting, diarrhea, fatigue, congestion, or one of the following: cough, shortness of breath, difficulty breathing, olfactory disorder, taste disorder, confusion, persistent chest pain, pale, gray, hypoxia, clinical or radiographic evidence of pneumonia or respiratory distress syndrome.	• Prematurity (gestational age less than 37 weeks)<br>• Anemia<br>• Immunodeficiency<br>• Cardiac abnormalities<br>• Pulmonary diseases<br>• Down syndrome<br>• Low birth weight (< 2,500 grams)	• Difficulty breathing<br>• Cough<br>• Croup<br>• Pain in throat / sore throat<br>• Nasal congestion<br>• Acute obstructive laryngitis<br>• Laryngeal stridor<br>• Pharyngitis<br>• Nasopharyngitis<br>• Otitis media<br>• Reactive airway diseases<br>• Upper respiratory tract infections recorded problem list with or without specifying pathogen	• Wheezing<br>• Bronchiolitis<br>• Bronchospasm<br>• Laryngotracheobronchitis<br>• Acute chest syndrome<br>• Pneumonia<br>• Hypoxia<br>• Hypoxemia<br>• Lower respiratory tract infections recorded in the problem list with or without specifying pathogen

One item from the column of URTI is required to meet the case definition of URTI; one item from the column of LRTI is required to meet the case definition of LRTI.

© 2025 Xu H et al. *JAMA Network Open*

eMethods. Data Abstraction From Electronic Medical Records

Data were abstracted from each patients' medical records electronically with support from Yale's Joint Data Analytics team. To identify acute respiratory illness (ARI) or relevant comorbidities, we searched administrative billing records, patient problem lists, and encounter diagnoses using SNOMED-CT or ICD-10-CM codes, supplemented by free-text searches to capture unstructured clinical details. To ensure reliability and validity, we manually reviewed 20% of records—focusing on those with missing key variables (e.g., encounter diagnosis, birth weight, gestational age, medical history)—to reconcile any inconsistencies and verify that risk factors were accurately recorded. A comprehensive list of the SNOMED codes and study terms used can be found in the study's online (GitHub) repository.

© 2025 Xu H et al. *JAMA Network Open*

eTable 2. Variable Selection for the Multivariate Logistic Regression Models

Outcome	Confounders	AIC
Medically attended RSV infection	age_tested[a] + month_tested + race_ethnicity + atleastone_risk_factor + insurance_type	2,853.0
	- race_ethnicity	2,849.0
	- insurance_type	2,846.8
	- atleastone_risk_factor (Final model: age_tested + month_tested)	2,845.1
RSV-associated outpatient visit	age_tested + month_tested + race_ethnicity + atleastone_risk_factor + insurance_type	2,242.0
	- race_ethnicity	2,238.1
	- insurance_type	2,236.3
	- atleastone_risk_factor (Final model: age_tested + month_tested)	2,235.6
RSV-associated hospitalization	age_tested + month_tested + race_ethnicity + atleastone_risk_factor + insurance_type + hospital	556.8
	- race_ethnicity	553.6
	- hospital	552.1
	- insurance_type (Final model: age_tested + month_tested + atleastone_risk_factor)	550.4
RSV-associated severe outcomes	age_tested + month_tested + race_ethnicity + atleastone_risk_factor + insurance_type + hospital	428.3
	- race_ethnicity	422.4
	- hospital	419.5
	- insurance_type (Final model: age_tested + month_tested + atleastone_risk_factor)	419.0
RSV-associated LRTI	age_tested + month_tested + race_ethnicity + atleastone_risk_factor + insurance_type	836.7
	- race_ethnicity	830.7
	- insurance_type (Final model: age_tested + month_tested + atleastone_risk_factor)	829.4
RSV-associated LRTI hospitalization	age_tested + month_tested + race_ethnicity + atleastone_risk_factor + insurance_type + hospital	300.7
	- hospital	297.7
	- race_ethnicity	293.9
	- insurance_type (Final model: age_tested + month_tested + atleastone_risk_factor)	293.0

The first row for each outcome presents the full model with all potential confounders. In each subsequent row, one variable is removed per step, with the final row showing the confounders included in the final model. Immunization status was included a priori in all models, and the final model was selected based on the lowest Akaike information criterion (AIC) score.

[a] Age when being tested (age_tested) are categorical variable in three-month interval.

© 2025 Xu H et al. *JAMA Network Open*

eFigure 2. Trace Plots for the Coefficients of Waning Effectiveness

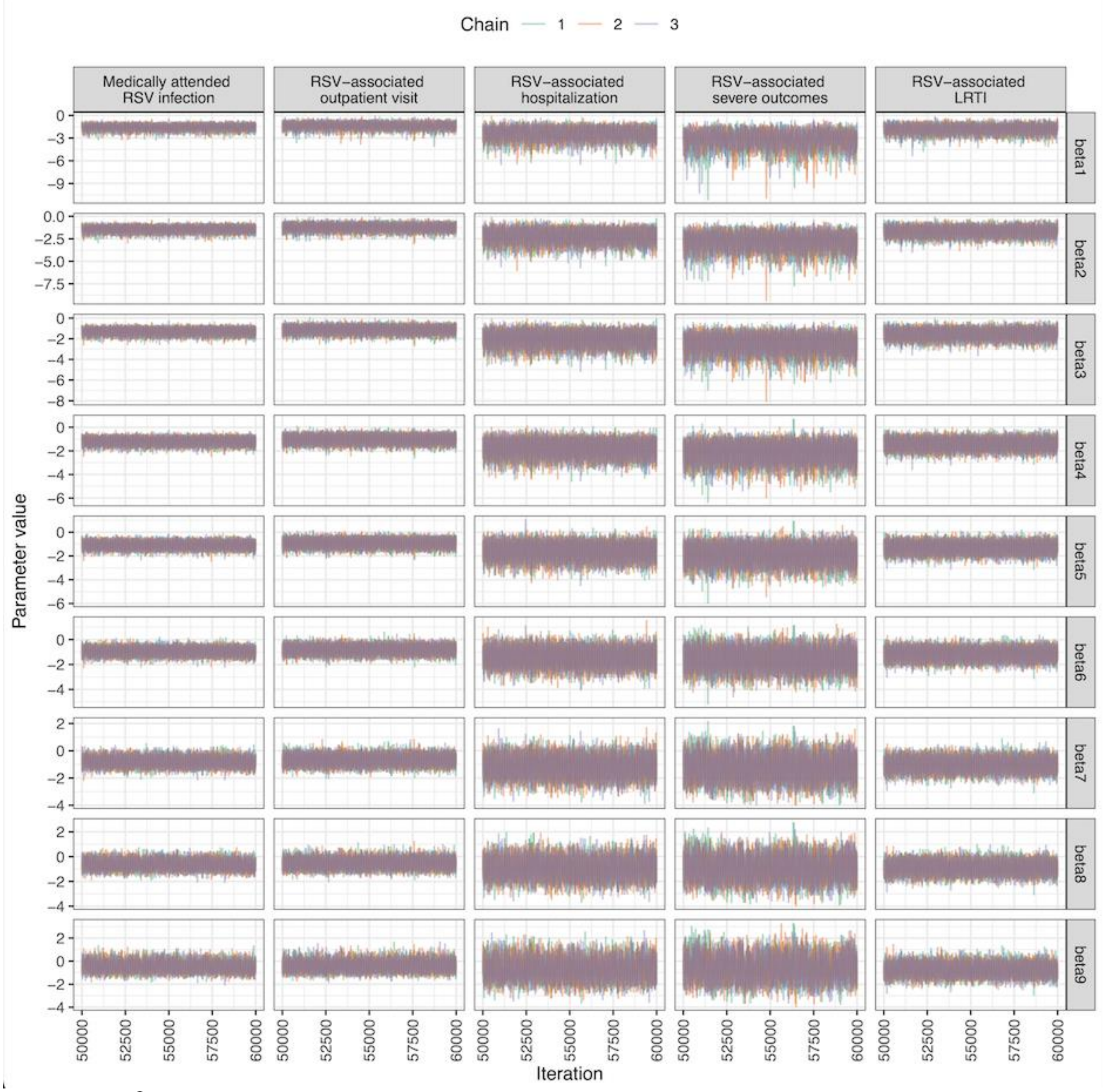

Trace plots for β_n (effectiveness coefficient for each biweek interval after nirsevimab immunization, n = 1,2, 3…9) are displayed by the examined outcome. All parameters demonstrate good convergence. Iterations of the burn-in period (iterations 0–50,000) are excluded, and only the sampled iterations (50,000–60,000) are presented.

© 2025 Xu H et al. *JAMA Network Open*

eAppendix. Estimating the Effectiveness of Nirsevimab by Time Since Immunization

We evaluated the waning of nirsevimab's protective effect over time since immunization using a logistic regression model within a Bayesian framework. For the jth test record in our dataset, the observed case status (i.e., whether the patient tested positive or negative for RSV) followed a Bernoulli distribution, such that

$$Case\_Status_j \sim Bernoulli(p_j).$$

The time between vaccination with nirsevimab and sample collection for RSV testing was categorized into nine bi-weekly intervals (2, 4, 6, ... >16 weeks). To incorporate this variable in the regression model, we created dummy variables to represent each time category ($time\_since\_vax_{jn}, n = 1,2,3 \ldots 9$). For example, if an individual was vaccinated 0-2 weeks before testing, $time\_since\_vax_{j1} = 1$ and $time\_since\_vax_{jn} = 0$ for $n = 2,3, \ldots 9$. For an unvaccinated individual, all the dummy variables $time\_since\_vax_{jn}$ ($n = 1,2,3, \ldots 9$) took the value of 0. The probability of testing positive, p_j, was modeled using a multivariate logistic regression framework as follows:

$$logit(p_j) = \beta_0 + \sum_{n=1}^{N} \beta_n time\_since\_vax_{jn} + \sum_{m=1}^{M} \gamma_m Z_{jm}$$

where Z_{jm} represents potential confounders (e.g., age at testing, month of testing, risk factors), and γ_m is the coefficients for the confounders.

Due to the waning nature of passive immunity, we assumed that nirsevimab's effectiveness had a non-increasing trend over time. To reflect this in the model, we imposed a monotonic structure on the regression coefficients β_n's, such that

$$\beta_{n+1} = \beta_n + d_{n+1}, n = 1, ,3, \ldots 9$$

$$d_{n+1} \sim Normal(0, \sigma_d^2)T(0,)$$

$$\sigma_d^2 \sim Inverse\ Gamma(0.01, 0.01)$$

$T(0,)$ represents truncation at 0, allowing d_{n+1} to take only non-negative values. For β_1 (coefficient for the effectiveness 0-2 weeks after vaccination), we used a weakly informative prior distribution:

$$\beta_1 \sim Normal(0, 100^2)$$

We examined effectiveness over time against various clinical endpoints. The model for each endpoint was fitted separately in the rjags package in R version 4.3.1, in which we collected 10,000 samples from the posterior distribution after discarding the first 50,000 samples in the burn-in period. Convergence was evaluated using trace plots (eFigure 7). The estimated effectiveness of nirsevimab after a given period of time (for time interval n) since immunization IE_n was calculated as

$$IE_n = \left(1 - e^{\beta_n}\right) * 100\%, n = 1,2, \ldots 9$$

Medians and 95% quantile-based credible intervals were calculated from the collected posterior samples.

© 2025 Xu H et al. *JAMA Network Open*

eTable 3. Sensitivity Analysis

	Exposure	Cases	Controls	Unadjusted effectiveness (95% CI)	Adjusted effectiveness (95% CI)
**Alternative Exposure Definitions[a]**					
Hepatitis B vaccine as 'sham' exposure	Immunized	365	1363	11 (-5.6-25)	-7 (-28.8-11)
	Unimmunized	315	1047		
Immunized ≤ 7 days before testing	Immunized	20	297	78.4 (66.7-86.8)	66.7 (47.2-80)
	Unimmunized	660	2113		
**Alternative Case Definition[b]**					
RSV-associated LRTI[c]	Immunized	11	57	82.1 (66.6-91.2)	71.7 (42-87.1)
	Unimmunized	350	325		
RSV-associated LRTI hospitalization[c]	Immunized	5	22	81.5 (53.3-94)	67.4 (-2.3-90.9)
	Unimmunized	145	118		
**Alternative Control Definition**					
Only positive for other viruses as controls[d]	Immunized	21	27	79.7 (63.3-88.9)	78.2 (54.8-89.7)
	Unimmunized	659	172		
**Alternative Sample Selection**					
Exclude infants with pre-existing immunity[e]	Immunized	17	247	77.3 (63.3-86.9)	66.6 (44.6-81)
	Unimmunized	265	874		
Exclude infants missing gestational age and birth weight	Immunized	19	289	78.6 (66.5-87.1)	68.5 (49.2-81.5)
	Unimmunized	476	1549		
**Alternative model: LASSO regression model[f]**					
Medically attend RSV infection	Immunized	21	309		60.8 (45.2-73.1)
	Unimmunized	659	2101		
RSV-associated outpatient visit	Immunized	16	231		59.2 (34.1-75.1)
	Unimmunized	498	1760		
RSV-associated hospitalization	Immunized	5	70		71.9 (49.1-86)
	Unimmunized	161	307		
RSV-associated severe outcomes	Immunized	4	78		80.2 (50.2-92.4)
	Unimmunized	141	212		
**Alternative model: Generalized Estimating Equation (GEE) model with pre-specified covariates[g]**					
Medically attended RSV infection	Immunized	21	309	77.7 (65.2-85.7)	67.6 (47.5-80.0)
	Unimmunized	659	2101		
RSV-associated outpatient visit	Immunized	16	231	74.1 (57.1-84.4)	61.5 (33.3-77.8)
	Unimmunized	498	1760		
RSV-associated hospitalization	Immunized	5	70	86.3 (65.1-94.7)	80.4 (46.4-92.9)
	Unimmunized	161	307		
	Immunized	4	78	92.2 (78.3-97.2)	84.4 (51.8-95)

© 2025 Xu H et al. *JAMA Network Open*

RSV-associated severe outcomes	Unimmunized	141	212		

[a] Effectiveness against medically attended RSV infection, adjusted for age tested and calendar month.
[b] Effectiveness against RSV-associated LRTI or LRTI hospitalization, adjusted for age tested, calendar month, and having at least one risk factor.
[c] Lower respiratory tract infection (LRTI): see definition in eTable 1.
[d] Other viruses: Influenza A, influenza B, adenovirus, rhinovirus, and parainfluenza.
[e] Excluded infants whose mother was immunized by RSV maternal vaccine and infants possibly exposed during the last RSV season (born before July 1st, 2023).
[f] A same set of pre-specified covariates was used in the penalized LASSO regression model as above.
[g] GEE model accounts for multiple tests obtained from the same individual, adjusting for multiple test results per person. Pre-specified covariates include age when being tested (categorical variable in three-month intervals), calendar month when being tested, race and ethnicity, having at least one risk factor, and type of insurance the patient had.

eFigure 3. RSV Tests and Nirsevimab Doses During the Study Period

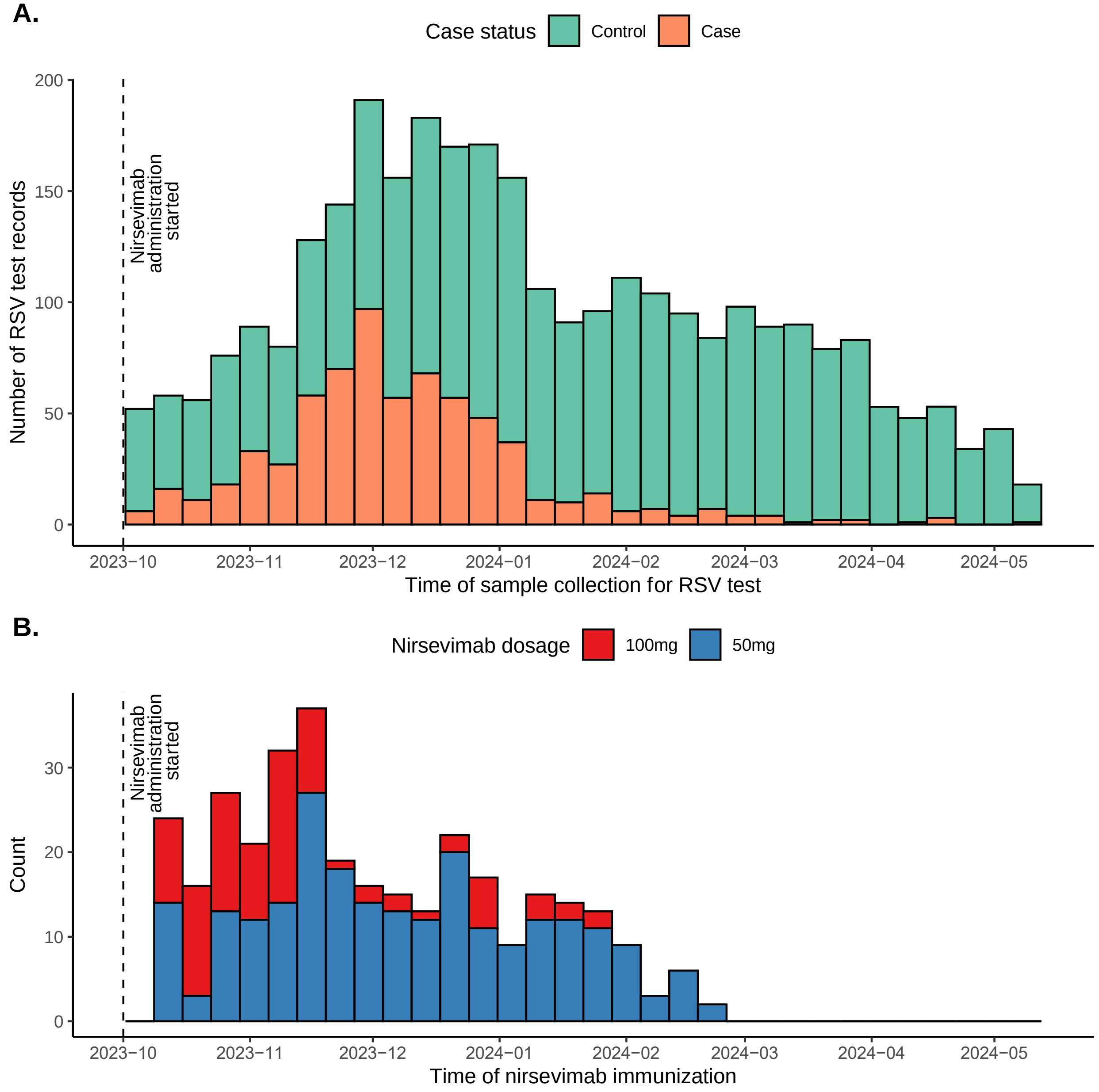

Panel A shows the number of RSV tests, with orange bars for RSV-positive cases and green bars for RSV-negative controls. Panel B displays the number of nirsevimab doses administered, with different colors of bars representing nirsevimab dosage. The vertical dashed line marks the start of nirsevimab administration in Connecticut (October 1, 2023).

eTable 4. Comparison of Included Records by Hospital Visited

Characteristic	Overall, N = 3,090[a]	Bridgeport Hospital, N = 950[a]	Greenwich Hospital, N = 399[a]	Lawrence + Memorial Hospital, N = 293[a]	Westerly Hospital, N = 80[a]	Yale New Haven Hospital, N = 1,368[a]	p-value[b]
Nirsevimab immunization							<0.001
Immunized	330 (10.7%)	76 (8.0%)	9 (2.3%)	38 (13.0%)	7 (8.8%)	200 (14.6%)	
Unimmunized	2,760 (89.3%)	874 (92.0%)	390 (97.7%)	255 (87.0%)	73 (91.3%)	1,168 (85.4%)	
RSV test result							0.726
RSV negative	2,410 (78.0%)	751 (79.1%)	303 (75.9%)	224 (76.5%)	63 (78.8%)	1,069 (78.1%)	
RSV positive	680 (22.0%)	199 (20.9%)	96 (24.1%)	69 (23.5%)	17 (21.3%)	299 (21.9%)	
Hospital admission[c]	543 (17.6%)	147 (15.5%)	54 (13.5%)	19 (6.5%)	2 (2.5%)	321 (23.5%)	<0.001
ICU admission[c]	99 (3.2%)	0 (0.0%)	3 (0.8%)	0 (0.0%)	0 (0.0%)	96 (7.0%)	
Required highflow oxygen support	417 (13.5%)	80 (8.4%)	30 (7.5%)	22 (7.5%)	3 (3.8%)	282 (20.6%)	<0.001
Distress in URT	1,353 (43.8%)	424 (44.6%)	168 (42.1%)	159 (54.3%)	30 (37.5%)	572 (41.8%)	0.002
Distress in LRT	743 (24.0%)	192 (20.2%)	92 (23.1%)	51 (17.4%)	19 (23.8%)	389 (28.4%)	<0.001
Fever (> 38°C/100.4°F)	1,419 (45.9%)	454 (47.8%)	239 (59.9%)	145 (49.5%)	36 (45.0%)	545 (39.8%)	<0.001
Cough	460 (14.9%)	141 (14.8%)	67 (16.8%)	41 (14.0%)	8 (10.0%)	203 (14.8%)	0.585
Wheezing	38 (1.2%)	5 (0.5%)	11 (2.8%)	2 (0.7%)	1 (1.3%)	19 (1.4%)	0.015
Breathing difficulties	48 (1.6%)	10 (1.1%)	1 (0.3%)	2 (0.7%)	2 (2.5%)	33 (2.4%)	0.004
Bronchiolitis	647 (20.9%)	171 (18.0%)	76 (19.0%)	44 (15.0%)	15 (18.8%)	341 (24.9%)	<0.001

[a]n (%)
[b]Pearson's Chi-squared test; Fisher's exact test
[c]Only hospital admission and ICU admissions with an admission date within 14 days of the RSV test are presented here.

© 2025 Xu H et al. *JAMA Network Open*

eTable 5. Comparison of Immunized and Unimmunized Patients

Characteristic	Overall, N = 3,090	Immunized, N = 330	Unimmunized, N = 2,760	Standardized Mean Difference[a]
Sex				0.03
Female	1,317 (42.6%)	138 (41.8%)	1,179 (42.7%)	
Male	1,772 (57.3%)	192 (58.2%)	1,580 (57.2%)	
Missing	1 (0.0%)	0 (0.0%)	1 (0.0%)	
Age at testing (months)				-0.84
Median (IQR)	6.7 (3.6, 9.7)	3.4 (1.8, 6.0)	7.1 (4.1, 10.0)	
Race and ethnicity				0.32
Hispanic	1,328 (43.0%)	138 (41.8%)	1,190 (43.1%)	
White non-Hispanic	820 (26.5%)	68 (20.6%)	752 (27.2%)	
Black non-Hispanic	533 (17.2%)	86 (26.1%)	447 (16.2%)	
Other non-Hispanic[b]	161 (5.2%)	24 (7.3%)	137 (5.0%)	
Unknown	248 (8.0%)	14 (4.2%)	234 (8.5%)	
Hospital visited				0.52
Bridgeport Hospital	950 (30.7%)	76 (23.0%)	874 (31.7%)	
Greenwich Hospital	399 (12.9%)	9 (2.7%)	390 (14.1%)	
Lawrence + Memorial Hospital	293 (9.5%)	38 (11.5%)	255 (9.2%)	
Westerly Hospital	80 (2.6%)	7 (2.1%)	73 (2.6%)	
Yale New Haven Hospital	1,368 (44.3%)	200 (60.6%)	1,168 (42.3%)	
Birth weight				-0.3
Median (IQR)	3,214.3 (2,824.9, 3,563.8)	3,099.6 (2,575.6, 3,483.7)	3,234.4 (2,875.0, 3,573.7)	
Missing	783 (25.3%)	26 (7.9%)	757 (27.4%)	
Gestational age				0.63
Less than 37 weeks	418 (13.5%)	90 (27.3%)	328 (11.9%)	
37 weeks or more	1,915 (62.0%)	218 (66.1%)	1,697 (61.5%)	
Missing	757 (24.5%)	22 (6.7%)	735 (26.6%)	
Pulmonary diseases	156 (5.0%)	19 (5.8%)	137 (5.0%)	0.04
Cardiac diseases	152 (4.9%)	33 (10.0%)	119 (4.3%)	0.22
Anemia	94 (3.0%)	11 (3.3%)	83 (3.0%)	0.02
Having at least one risk factor[c]	750 (24.3%)	123 (37.3%)	627 (22.7%)	0.32
Insurance type				0.24
Private	983 (31.8%)	78 (23.6%)	905 (32.8%)	
Public	2,088 (67.6%)	252 (76.4%)	1,836 (66.5%)	
Uninsured	19 (0.6%)	0 (0.0%)	19 (0.7%)	

Data are presented as median (IQR) for continuous measures and n/total (%) for categorical measures.
[a] Standardized mean difference: the difference in means between case and control participants in units of the pooled SD. Covariates with an absolute standardized mean difference greater than 0.2 were considered to have important imbalances.
[b] Inclusing Asian, Pacific Islander, Middle Eastern or Northern American, American Indian, or Native American by self-reporting.
[c] Have at least one of the following conditions recorded in the infant's medical history or diagnosis records: 1) Anemia; 2) Immunodeficiency (e.g., transplantation history, leukemia, etc.); 3) Cardiac diseases (including congenital heart diseases diagnosed at birth or any reporting of heart conditions); 4) Pulmonary diseases; 5) Down syndrome; 6) Small for gestational age (birth weight < 2,500 grams); 7) Prematurity (gestational age less than 37 weeks).

© 2025 Xu H et al. *JAMA Network Open*

eTable 6. Clinical Characteristics of RSV-Positive Cases

Characteristic	**Overall, N = 680a**	**Unimmunized, N = 659a**	**Immunized**, N = 21[a]
Hospital admission	166 (24.4%)	161 (24.4%)	5 (23.8%)
Duration of hospitalization (days)			
Median (IQR)	1.0 (1.0, 2.0)	1.0 (1.0, 2.0)	3.0 (2.0, 3.0)
N missing (% missing)	514 (75.6%)	498 (75.6%)	16 (76.2%)
ICU admission	23 (3.4%)	22 (3.3%)	1 (4.8%)
Duration of ICU admission (days)			
Median (IQR)	3.1 (1.8, 7.3)	3.5 (1.8, 7.4)	2.4 (2.4, 2.4)
N missing (% missing)	657 (96.6%)	637 (96.7%)	20 (95.2%)
Required high-flow oxygen support	145 (21.3%)	141 (21.4%)	4 (19.0%)
Upper respiratory tract infection (URTI)[b]	229 (33.7%)	221 (33.5%)	8 (38.1%)
Lower respiratory tract infection (LRTI)[c]	361 (53.1%)	350 (53.1%)	11 (52.4%)
Fever (> 38°C/100.4°F)	239 (35.1%)	235 (35.7%)	4 (19.0%)
Cough	98 (14.4%)	94 (14.3%)	4 (19.0%)
Wheezing	9 (1.3%)	9 (1.4%)	0 (0.0%)
Breathing difficulties	3 (0.4%)	3 (0.5%)	0 (0.0%)
Bronchiolitis	349 (51.3%)	338 (51.3%)	11 (52.4%)
Sepsis	2 (0.3%)	2 (0.3%)	0 (0.0%)

[a] Data are presented as median (IQR) for continuous measures and n/total (%) for categorical measures.

[b,c] See eTable 1 for definitions of URTI and LRTI.

© 2025 Xu H et al. *JAMA Network Open*

eFigure 4. Overview of Nirsevimab Effectiveness: Current Study Estimates in Context With Prior Research

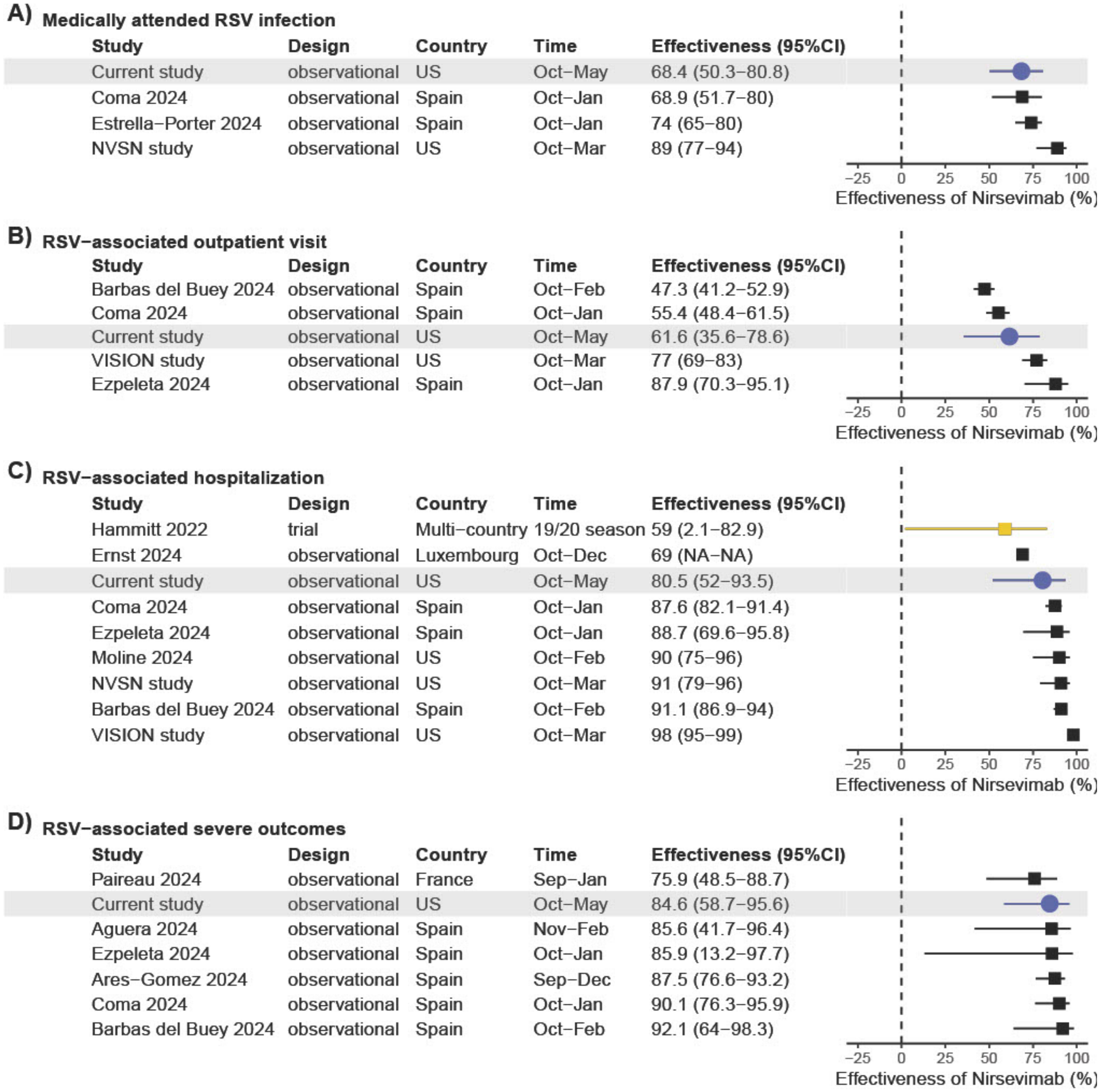

This figure contrasts adjusted effectiveness (post-licensure) and efficacy (pre-licensure) estimates from prior studies with those from the current study. The right panel shows means (dots) and uncertainty intervals (bars). Gold squares represent Phase IIb/III trial data, black squares represent observational studies, and blue circles represent the current study (highlighted in gray). Ernst et al. 2024[1] did not report uncertainty intervals. Studies included in the above figures are listed in the references section[1–11].

eFigure 5. Effectiveness of Nirsevimab Against RSV Infections by Dose, Clinical Setting, and Disease Severity

Outcome prevented	RSV positive	RSV negative	Unadjusted Effectiveness (95%CI)	Adjusted Effectiveness (95%CI)
**Medically attended RSV infection**				
Not immunized	659	2101	REF	REF
Immunized (50mg)	15	220	78.3 (64.3−87.7)	66 (42.1−81.4)
Immunized (100mg)	6	89	78.5 (54.7−91.7)	72.7 (40.6−89.6)
Immunized (any dosage)	21	309	78.3 (66.8−86.6)	68.4 (50.3−80.8)
**RSV−associated outpatient visit**				
Not immunized	498	1760	REF	REF
Immunized (50mg)	11	161	75.9 (57.2−87.7)	58.1 (21.5−79.5)
Immunized (100mg)	5	70	74.8 (43.1−91.2)	67.1 (22.9−88.8)
Immunized (any dosage)	16	231	75.5 (60.3−86)	61.6 (35.6−78.6)
**RSV−associated hospitalization**				
Not immunized	161	307	REF	REF
Immunized (50mg)	4	51	85 (62.6−95.5)	77.8 (39.6−93.7)
Immunized (100mg)	1	19	90 (51−99.4)	87.9 (34.3−99.4)
Immunized (any dosage)	5	70	86.4 (68.7−95.3)	80.5 (52−93.5)
**RSV−associated severe outcomes**				
Not immunized	141	212	REF	REF
Immunized (50mg)	3	60	92.5 (79.2−98.2)	84.7 (52.9−96.5)
Immunized (100mg)	1	18	91.6 (58.9−99.5)	85.8 (17.9−99.3)
Immunized (any dosage)	4	78	92.3 (80.9−97.7)	84.6 (58.7−95.6)

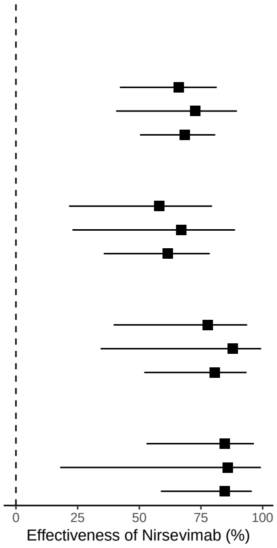

Square dots indicate mean effectiveness estimates, with horizontal lines representing 95% confidence intervals. All models adjusted for age and calendar month. Models for RSV-associated hospitalization and severe disease also accounted for the presence of underlying risk factors. Only hospitalizations and ICU admissions with an admission date within 14 days of RSV testing were included in the analysis.

© 2025 Xu H et al. *JAMA Network Open*

eFigure 6. Effectiveness of Nirsevimab Against RSV-Associated LRTI by Time Since Immunization

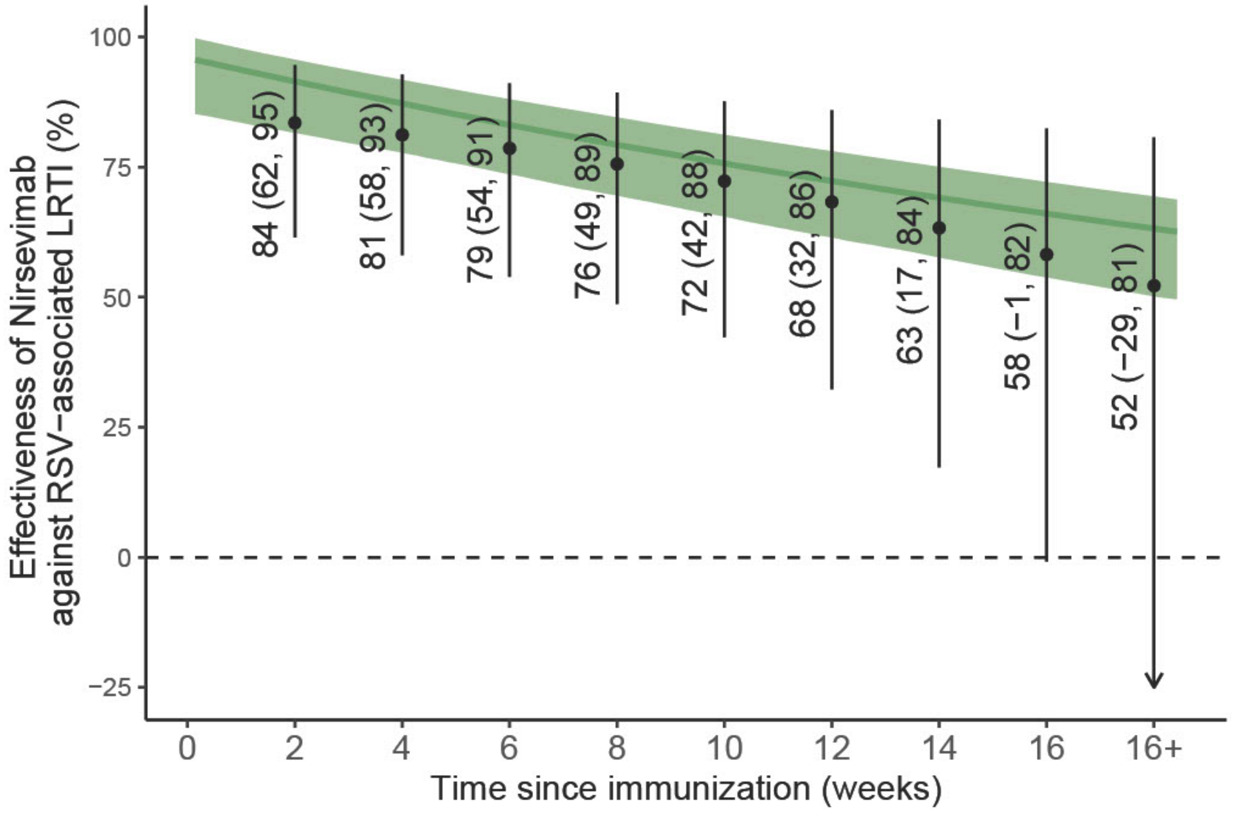

The green curve and shaded area represent the median and 95% credible interval of the estimated efficacy of nirsevimab reported by Hodgson et al.[12], where efficacy over time was estimated using data from Phase IIb and Phase III trials in a survival model. The black dots denote the median estimates of nirsevimab effectiveness in preventing RSV-associated LRTI from our current study, using the same endpoint as in Hodgson et al. for comparison[12]. The error bars show the 95% credible intervals for the estimates, and the labels provide the exact values.

eFigure 7. Effectiveness of Nirsevimab Against Medically Attended RSV Infection Over Calendar Time

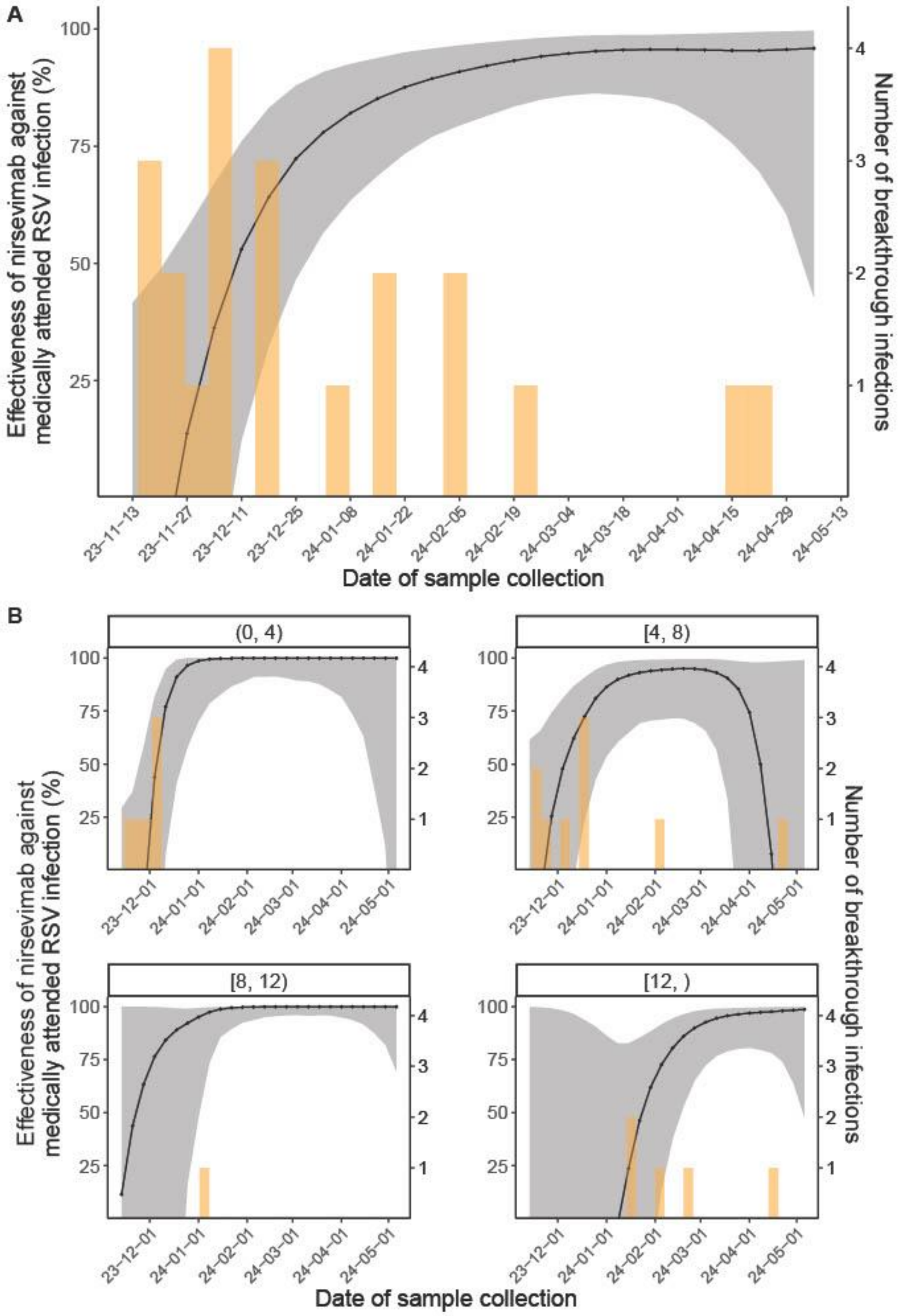

The solid lines and gray ribbons represent the median and 95% credible intervals of effectiveness over calendar time, estimated using INLA models with fixed and random effects for nirsevimab effectiveness, incorporating second-order random walk smoothing. Orange bars indicate the number of breakthrough infections over time. In panel (B), effectiveness is further stratified by time since immunization, shown in four sub-panels: 0–4 weeks, 4–8 weeks, 8–12 weeks, and ≥12 weeks before RSV testing.

© 2025 Xu H et al. *JAMA Network Open*

eFigure 8. Subgroup Analysis of Nirsevimab Effectiveness Against Medically Attended RSV Infections

	Immunized	Unimmunized	Effectiveness (95%CI)
**Having at least one risk factor**			
Yes	3/123	147/627	78.2 (56.5, 89.4)
No	18/207	512/2133	49.8 (26.7, 69)
**Age when being tested**			
Under 6 months	17/247	312/1112	64.1 (45.9, 78.6)
6 months and above	4/83	347/1648	55.5 (10.6, 83)
**Ethnicity**			
Hispanic or Latino	10/138	270/1190	67.2 (46.4, 85)
Not Hispanic or Latino	10/180	333/1372	63.4 (34.6, 79.8)
**Race**			
White	9/117	280/1105	52.7 (21.9, 80.7)
Black	3/100	144/577	83 (64.7, 96)
**Month tested**			
Peak (Dec.−Jan.)	10/121	323/1125	73.8 (57.3, 86.5)
Off−peak (other months)	11/209	336/1635	72.2 (58, 84.8)
**Insurance type**			
Private	6/78	225/905	38 (0, 67.5)
Public or uninsured	15/252	434/1855	69.5 (51.4, 82.6)
**Gestational age**			
Less than 37 weeks	3/90	73/328	66.7 (41.2, 82.9)
37 weeks and more	16/218	403/1697	60.1 (35, 75.5)

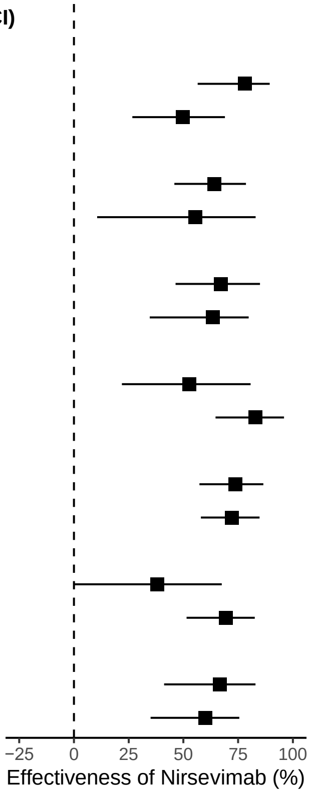

This figure presents results from a post hoc sensitivity analysis testing for potential effect modification. Each square marker represents the estimated effectiveness of nirsevimab against medically attended RSV infections for a given subgroup, with horizontal lines indicating the corresponding 95% confidence intervals. Effectiveness estimates were derived using penalized (LASSO) logistic regression, with interaction terms for age at testing (categorized in six-month intervals), testing calendar month, race, ethnicity, presence of at least one risk factor, and insurance type.

© 2025 Xu H et al. *JAMA Network Open*

eREFERENCES

1. Ernst C, Bejko D, Gaasch L, et al. Impact of nirsevimab prophylaxis on paediatric respiratory syncytial virus (RSV)-related hospitalisations during the initial 2023/24 season in Luxembourg. *Euro Surveill*. 2024;29(4). doi:10.2807/1560-7917.ES.2024.29.4.2400033

2. Ezpeleta G, Navascués A, Viguria N, et al. Effectiveness of nirsevimab immunoprophylaxis administered at birth to prevent infant hospitalisation for respiratory syncytial virus infection: A population-based cohort study. *Vaccines (Basel)*. 2024;12(4):383.

3. Hammitt LL, Dagan R, Yuan Y, et al. Nirsevimab for Prevention of RSV in Healthy Late-Preterm and Term Infants. *N Engl J Med*. 2022;386(9):837-846.

4. Summary of effectiveness of nirsevimab in infants. 2024. https://www.cdc.gov/vaccines/acip/meetings/downloads/slides-2024-06-26-28/04-RSV-Mat-Peds-Payne-508.pdf

5. Estrella-Porter P, Blanco-Calvo C, Lameiras-Azevedo AS, et al. Effectiveness of nirsevimab introduction against respiratory syncytial virus in the Valencian Community: A preliminary assessment. *Vaccine*. 2024;42(22):126030.

6. Coma E, Martinez-Marcos M, Hermosilla E, et al. Effectiveness of nirsevimab immunoprophylaxis against respiratory syncytial virus-related outcomes in hospital and primary care settings: a retrospective cohort study in infants in Catalonia (Spain). *Arch Dis Child*. 2024;109(9):736-741.

7. Barbas Del Buey JF, Íñigo Martínez J, Gutiérrez Rodríguez MÁ, et al. The effectiveness of nirsevimab in reducing the burden of disease due to respiratory syncytial virus (RSV) infection over time in the Madrid region (Spain): a prospective population-based cohort study. *Front Public Health*. 2024;12:1441786.

8. Moline HL. Early Estimate of Nirsevimab Effectiveness for Prevention of Respiratory Syncytial Virus–Associated Hospitalization Among Infants Entering Their First Respiratory Syncytial Virus Season—New Vaccine Surveillance Network, October 2023–February 2024. *MMWR Morb Mortal Wkly Rep*. 2024;73.

9. Paireau J, Durand C, Raimbault S, et al. Nirsevimab Effectiveness Against Cases of Respiratory Syncytial Virus Bronchiolitis Hospitalised in Paediatric Intensive Care Units in France, September 2023-January 2024. *Influenza Other Respi Viruses*. 2024;18(6):e13311.

10. Agüera M, Soler-Garcia A, Alejandre C, et al. Nirsevimab immunization's real-world effectiveness in preventing severe bronchiolitis: A test-negative case-control study. *Pediatr Allergy Immunol*. 2024;35(6):e14175.

11. Ares-Gómez S, Mallah N, Santiago-Pérez MI, et al. Effectiveness and impact of universal prophylaxis with nirsevimab in infants against hospitalisation for respiratory syncytial virus in Galicia, Spain: initial results of a population-based longitudinal study. *Lancet Infect Dis*. Published online 2024.

12. Hodgson D, Wilkins N, van Leeuwen E, et al. Protecting infants against RSV disease: an impact and cost-effectiveness comparison of long-acting monoclonal antibodies and maternal vaccination. *Lancet Reg Health Eur*. 2024;38:100829.
