# Supplementary material for: Estimated Effectiveness of Nirsevimab Against Respiratory Syncytial Virus
Source: JAMA Netw Open. 2025 Mar 10;8(3):e250380. doi: 10.1001/jamanetworkopen.2025.0380 (PMC11894488; doi:10.1001/jamanetworkopen.2025.0380)
Supplement: Supplement 2. — Data Sharing Statement [file jamanetwopen-e250380-s002.pdf]

Data Sharing Statement

Xu. Effectiveness of Nirsevimab Against Respiratory Syncytial Virus. *JAMA Netw Open*. Published March 10, 2025. doi:10.1001/jamanetworkopen.2025.0380

Data
Data available: Yes
Data types: Data dictionary, Deidentified participant data
How to access data: External researchers can make written requests to the corresponding author for sharing of completely de-identified and aggregate-level data. The study protocol and statistical code used are also available on github repository.
When available: With publication

Supporting Documents
Document types: Statistical/analytic code
How to access documents: https://github.com/Hanmeng-Xu/RSV_mAb_VE
When available: With publication

Additional Information
Who can access the data: Data are available for researchers provided all ethical and legal requirements are met.
Types of analyses: To allow replication of results.
Mechanisms of data availability: Requests will be assessed on a case-by-case basis in consultation with the lead and co-investigators. All data sharing will abide by rules and policies defined by the involved parties. Data-sharing mechanisms will ensure that the rights and privacy of individuals participating in research will be protected at all times.
